# Pulmonary Arterial Hypertension: Iron Matters

**DOI:** 10.3389/fphys.2018.00641

**Published:** 2018-05-31

**Authors:** Latha Ramakrishnan, Sofia L. Pedersen, Quezia K. Toe, Gregory J. Quinlan, Stephen J. Wort

**Affiliations:** Cardiorespiratory Interface – Vascular Biology, The National Heart and Lung Institute, Faculty of Medicine, Imperial College London, London, United Kingdom

**Keywords:** iron, hepcidin and ferroportin 1 (Fpn1), pulmonary arterial hypertension, pulmonary arterial remodeling, pulmonary hypertension

## Abstract

The interplay between iron and oxygen is longstanding and central to all aerobic life. Tight regulation of these interactions including homeostatic regulation of iron utilization ensures safe usage of this limited resource. However, when control is lost adverse events can ensue, which are known to contribute to an array of disease processes. Recently, associations between disrupted iron homeostasis and pulmonary artery hypertension (PAH) have been described with the suggestion that there is a contributory link with disease. This review provides a background for iron regulation in humans, describes PAH classifications, and discusses emerging literature, which suggests a role for disrupted iron homeostatic control in various sub-types of PAH, including a role for decompartmentalization of hemoglobin. Finally, the potential for therapeutic options to restore iron homeostatic balance in PAH are discussed.

## Background of Iron Handling in Health

### Iron and Oxygen

Iron is the principal catalyst that allows for oxygen utilization. The electronic structure of ground state molecular oxygen provides inherent stability (two unpaired electrons with parallel spin); so called spin restriction. Ground state molecular oxygen is, therefore, a relatively unreactive molecule. In order to facilitate oxygen utilization for metabolism, conversion to a reactive state (activation) is achieved via single electron transfer reactions. Iron, as a classical transition metal, has the ability to exist in different states of valence and, therefore, the ability to donate or accept electrons singly, enabling it to convert oxygen to a reactive and therefore metabolically active state. Consequently, body iron requirements are almost exclusively involved with some aspect of oxygen utilization. Notable examples include: respiration, molecular transport, molecular storage, antioxidant protection and biosynthesis.

### Mammalian Iron Requirements

Healthy human adults contain between 2 and 4 g of iron; daily iron requirements for metabolism and biosynthesis are 20 mg, largely for heme biosynthesis, to satisfy the daily requirement for the production of 200 billion red blood cells. However, iron utilization is not limited to these processes; for instance, all cells require iron to proliferate, iron being essential for DNA biosynthesis as well as for cell cycle progression (Yu et al., 2007). In addition, many proteins and particularly those involved in oxygen metabolism have an essential requirement for iron, which is usually localized to heme and non- heme containing active centers. Mitochondria are principal cellular sites for heme and iron–sulfur cluster biosynthesis and therefore require an adequate supply of iron to maintain these activities.

Iron uptake from the diet is largely facilitated by enterocytes localized to the duodenum but these can provide only 1–2 mg of iron on a daily basis. Moreover, daily iron losses are similar; although no specific iron excretory mechanisms exist in mammals, losses do occur through shedding of intestinal epithelial cells and skin cells, blood loss and, in addition, via bile and urine excretion. Iron uptake from the gut therefore balances these losses but cannot accommodate the daily requirement of 20 mg. The majority of this essential iron requirement is therefore supplied by recycling endogenous iron resources and stores rather than by intestinal uptake, and is under strict regulatory control, not least because iron is a limited and precious resource.

### Iron Homeostatic Control

#### Cellular Regulation

Regulation of cellular iron requirements are chiefly facilitated by post-transcriptional feedback mechanisms directed by the activity of two cytosolic iron regulatory proteins, IRP-1 and IRP-2, which, when active, bind to key regulatory motifs termed iron responsive elements (IREs) either located at the 5^′^ or 3^′^ ends in target mRNAs. IRP binding to 5^′^ IREs prevents ribosomal translation and hence biosynthesis, whereas 3^′^ binding stabilizes mRNA and supports translation. IRPs are activated by low cytosolic cellular iron levels. Under these circumstances synthesis of both light (L) and heavy (H) chains of the intracellular iron storage protein complex, ferritin, is down regulated (as is the synthesis of the transmembrane protein and iron exporter, ferroportin). Importantly, the translation of hypoxia inducible factor (Hif)-2α which is one component of the Hif complex, is also inhibited demonstrating the interplay between iron and oxygen homeostasis (see also section “Oxygen Sensing and Iron Regulation”). Conversely, IRP 3’ IRE mRNA binding promotes the synthesis of transferrin receptor 1 (TFR-1), a transmembrane glycoprotein that facilitates the uptake of iron-loaded transferrin from the circulation via receptor mediated endocytosis. Additionally, divalent metal transporter 1 (DMT-1) a protein that binds to a variety of metals including cadmium, copper, zinc, and iron, provides an additional route for (direct) iron uptake by cells. Thus, in situations when cellular iron levels are low, active IRPs down-regulate iron storage and cellular export whilst facilitating cellular iron uptake. The opposite occurs when cellular iron levels are replete or overloaded- IRPs are inactivated. Operational within cells such as enterocytes, macrophages, and hepatocytes, and important for iron turnover and control, it is also apparent that such regulation is common to other cell types. In addition, IREs have been identified in mRNAs for numerous proteins beyond those described above indicating a more complex role for the IRPs in cellular regulation of iron and oxygen homeostasis; for reviews, see Kuhn (2015) and Simpson and McKie (2015).

#### Global Regulation: The Importance of the Hepcidin-Ferroportin Axis

**FIGURE 1:**
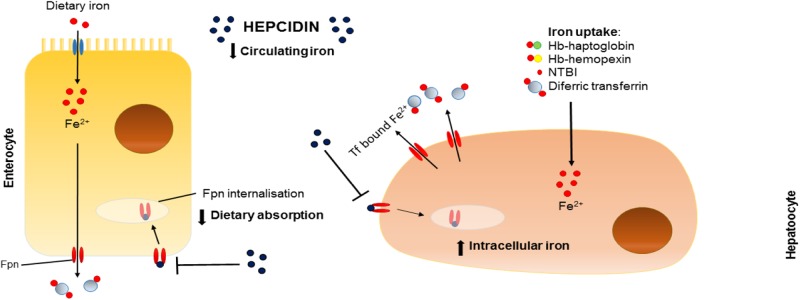
Effects of hepcidin on iron homeostasis. Schematic representation of the effects of hepcidin on dietary absorption of iron (left) and intracellular iron stores (right). Fpn, ferroportin; Tf, transferrin; Fe, iron; Hb, hemoglobin; NTBI, non-transferrin-bound iron.

Often described as the master regulator of iron homeostasis, hepcidin is a small peptide hormone (25 amino acids) synthesized in the main by liver hepatocytes; a process that is regulated by plasma and liver iron levels and which involves signaling via bone morphogenetic protein (BMP) and SMAD pathways, inflammation and, in particular, IL-6 levels via the JAK/stat pathway. In the circulation hepcidin targets and binds to cellular ferroportin causing it to be endocytosed and degraded hence halting cellular iron export (**Figure 1**).

**FIGURE 2:**
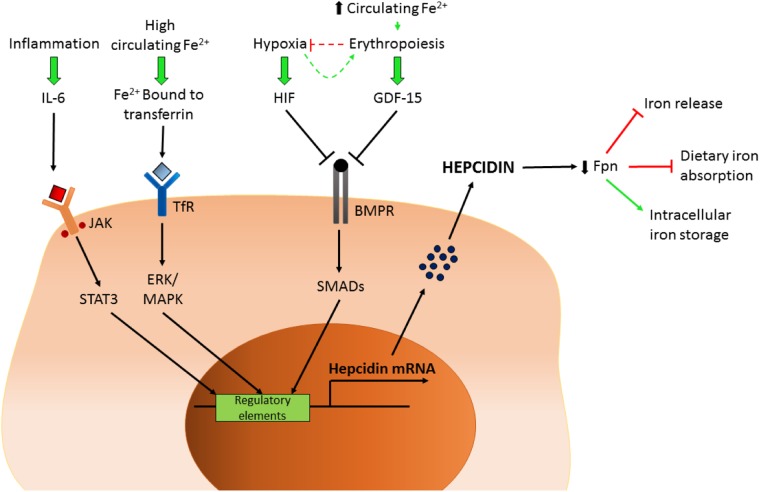
Control of hepcidin expression. Schematic representation of the factors controlling hepcidin transcription. IL-6, interleukin 6; JAK/STAT3, Janus kinase/signal transducers and activator of transcription 3; Fe, iron; TfR, transferrin receptor; HIF, hypoxia-inducible factor; GDF-15, growth/differentiation factor 15; BMPR, bone morphogenetic protein receptor; Fpn, ferroportin.

Hypoxia and erythropoiesis are also important regulatory signals for hepcidin production (**Figure 2**). Consequences of such inhibition include prevention of intestinal iron absorption, limitation of release of liver iron stores and hindrance of recycling processes linked to macrophages. If hepcidin release is sustained, the accumulation of iron in tissues (hepatocytes, macrophages, and other cell types) coupled with limited iron uptake from the diet result in a negative iron balance and tissue iron loading. For a comprehensive review, see Drakesmith et al. (2015).

#### Oxygen Sensing and Iron Regulation

Iron and oxygen utilization are closely linked and longstanding in nature, being essential for all aerobic life. This relationship is aptly illustrated by the joint regulatory roles for both oxygen and iron in the control of the activity of Hif. Hif is composed of an oxygen-dependent subunit, Hif 2α, and a constitutively expressed β subunit. The prolyl hydroylases (PHDs) hydroxylate two key prolyl residues on Hif 2α which ultimately leads to ubiquitination and degradation, so preventing HiF assembly and activation. Importantly, both oxygen and iron are required for enzyme activity of the PHDs. Conversely, when either iron or oxygen levels are low, transcription factor assembly occurs and binding to target hypoxia responsive elements in the promoter regions of genes regulated by Hif is facilitated. Hif is a multifunctional transcription factor involved in expression of genes linked to cytoskeleton formation, energy metabolism, and erythropoiesis and importantly, in the context of this review: vasomotor function, migration, proliferation, angiogenesis, and the regulation of iron transport. For a general review of Hif, see Simpson and McKie (2015).

## Evidence for Importance of Iron and Related Molecules in the Normal Human Vasculature

Literature describing the role of iron in the maintenance of balanced vascular function is somewhat limited with most studies on iron homeostasis focusing on global aspects involved in control of erythropoiesis. However, some studies undertaken with healthy human volunteers have demonstrated that iron chelation with desferrioxamine promotes hypoxic vasoconstriction (HPV) and increases pulmonary artery systolic pressure (PASP) compared to iron replete individuals (Smith et al., 2008). Furthermore, the same group performed two randomized placebo controlled trials investigating the effect of iron on HPV and PASP. In the first, a group of sea-level dwelling individuals were taken to altitude; iron infusion resulted in a 6 mmHg fall in the pulmonary hypertensive response initiated by hypoxia. In the second protocol, patients with chronic mountain sickness received isovolaemic 2 l venesections followed 2 weeks later by an infusion of iron or placebo. Venesection resulted in a 25% increase in PASP. However, subsequent iron infusion did not ameliorate the increase in PASP (Smith et al., 2009). Additional support for the role of iron in HPV is provided by Frise et al. (2016). In 13 iron deficient individuals, 6 h of hypoxia led to an increase in PASP compared to iron replete controls. Intravenous iron (given before the hypoxic challenge) attenuated the rise in PASP in both groups but to a greater extent in the iron deficient group (Frise et al., 2016). The above findings indicate a key role for iron in the sensing and signaling response to hypoxia in normal pulmonary vascular function.

As for evidence of any role for iron regulation at the level of the pulmonary vascular cell, these studies are somewhat lacking but new findings from our own laboratory have recently demonstrated the presence of the iron exporter ferroportin in pulmonary artery smooth muscle cells (PASMCs) and pulmonary artery endothelial cells (PAECs) (Ramakrishnan et al., 2014) suggesting a potential dynamic role for the hepcidin-ferroportin axis and the regulation of cellular iron stores at the level of the pulmonary vasculature. Please see Section “Group 3: PH Related to Chronic Lung Disease and/or Hypoxia (Including High Altitude)” for further discussion of response of the pulmonary vasculature to hypoxia and relevance to the development of pulmonary hypertension (PH).

## Pulmonary Hypertension: Definition and Classification

**FIGURE 3:**
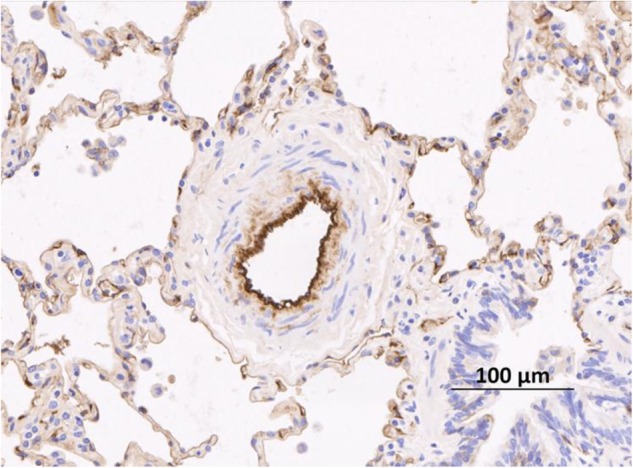
Remodeled pulmonary arteriole from a patient with idiopathic pulmonary arterial hypertension taken after transplant. Remodeling in this case is characterized by an increase in the number of smooth muscle cells in the media. The endothelium is stained with an anti-vWF antibody (brown stain). vWF, von Willibrand Factor. Figure courtesy of Dr. Allan Lawrie and Dr. Roger Thompson, University of Sheffield, United Kingdom.

Pulmonary hypertension encompasses a group of conditions characterized by raised blood pressure in the pulmonary arteries. The formal diagnosis requires right heart catheterization: PH is defined as a mean pulmonary arterial pressure ≥25 mmHg at rest. There is a further hemodynamic division into pre- and post- capillary PH depending on whether the pulmonary artery wedge pressure (a measure of left atrial pressure) is ≤15 mmHg (pre-capillary PH) or >15 mmHg (post-capillary PH). PH is divided into five clinical groups, each group of which shares similar pathophysiology and anticipated response to treatment (Galie et al., 2016). Group 1 PH is known as pulmonary arterial hypertension (PAH). All the conditions within this group have pre-capillary hemodynamics, a raised pulmonary vascular resistance (PVR) and no evidence of significant lung disease (Group 3 PH) or thromboembolic disease (Group 4). The main disorders presenting with PAH are congenital heart disease (predominantly Eisenmenger Syndrome), scleroderma associated PAH and idiopathic (i)PAH (a diagnosis of exclusion) (Humbert et al., 2006; Peacock et al., 2007). Within the clinical phenotype of Group 1 PAH, a small proportion will have a family history and most will carry a genetic abnormality in one of the genes associated with the condition, predominantly, bone morphogenetic protein receptor (BMPR) 2 (Rudarakanchana et al., 2002; Soubrier et al., 2013). All members of Group 1 have similar histology with remodeling of pulmonary arterioles (diameter < 500 μM). This involves hyperplasia of cells encompassing all three layers of the vessel wall, although predominantly smooth muscle (**Figure 3**). The resulting increase in PVR increases the afterload on the right ventricle (RV) provoking RV hypertrophy, enlargement and eventually failure (Tuder et al., 2013). PAH is always associated with increased morbidity and mortality, but with some heterogeneity depending on sub-type. Patients with iPAH have one of the worse prognoses with a pre-treatment era median survival of only 2.8 years, comparable to many advanced cancers (Barst et al., 1996). In addition, it has a female predominance and tends to affect younger adults (Peacock et al., 2007).

### Pathophysiology of PAH

This review is predominantly involved with the pre-capillary remodeling observed in PAH, although pure vasoconstriction is likely to be involved in acute responses to hypoxia, described in other sections.

The exact sequence of events leading to pulmonary vascular remodeling remains unknown; however, the lung pulmonary vasculature does show a stereotypical response to insult(s) as histopathology of lesions remains very similar across sub-types of PAH, see **Figure 3** for a representative remodeled human pulmonary arteriole On the one hand, it is very likely that increased endothelial shear stress from left to right blood flow across an intra- or extra-cardiac defect is the initiating insult in Eisenmenger syndrome (D’Alto and Mahadevan, 2012). On the other, in heritable PAH, defects predominantly in the BMPR2 gene increase susceptibility to developing PAH. However, the penetrance of the genetic defect remains low, suggesting other “hits” are necessary. Additional insults are likely to include: infection, exposure to hypoxia, exposure to serotoninergic drugs and pregnancy related changes in female hormone levels (Tuder et al., 2013). Although not proven, one of the earliest abnormalities is probably endothelial cell dysfunction leading to an imbalance of vasoactive molecules: increased production of the vasoconstrictor and mitogen, endothelin (ET)-1 and reduced production of nitric oxide (NO) and prostacyclin (PGI_2_), both vasodilators and anti-proliferative agents (Humbert et al., 2004). Damage to the endothelium may expose the underlying smooth muscle (SM) to cytokines and serum factors that promote proliferation.

The presence of BMPR2 mutations pre-dispose SM cells to increased proliferative rates and reduced apoptosis (Yang et al., 2011). Medial (SM) hypertrophy in resistance arterioles is one of the cardinal histological features of PAH (**Figure 3**). As the disease progresses, it is likely that apoptotic resistant endothelial cells lead to neointimal formation and fibrosis and the formation of plexiform lesions. The adventitia is also involved with increased numbers of fibroblasts and extra-cellular matrix protein deposition. Inflammatory changes are often observed surrounding remodeled vessels, although the exact contribution of inflammation (marker or mediator) remains unclear. However, circulating cytokines, such as IL-6, IL-8, and IL-10 are increased in patients with PAH and correlate with outcome (Soon et al., 2010). Other important mediators are growth factors such as platelet derived growth factor (PDGF) and transforming growth factor (TGF)- β. Most recently, it is clear that there are also epigenetic mechanisms involved (Pullamsetti et al., 2016). A more complete discussion, apart from reviewing the role of iron and iron-related molecules below is beyond the scope of the present review.

## Evidence for Abnormal Iron Handling in Pulmonary Hypertension

Most of the literature relating abnormal iron handling to the development of PH concentrates on iPAH and Eisenmenger syndrome, which will be the main focus of this section. However, there is good evidence to relate iron deficiency to an exaggerated response to hypoxia, which may of course relate to respiratory conditions associated with PH in Group 3 of the international classification as well as exposure to high altitude. Chronic exposure to hypoxia, and the development of high altitude PH has been the topic of several recent reviews (Wilkins et al., 2015). See also Section “Group 3: PH Related to Chronic Lung Disease and/or Hypoxia (Including High Altitude)” of this review.

### Evidence for Abnormal Iron Handling in iPAH

Ruiter et al. (2011) reported the first data to support iron deficiency in patients with iPAH. In 70 patients, 30 (43%) had iron deficiency as determined by a serum iron < 10μmol^-1^ and transferrin saturation < 15% in females and <20% in males; in normal individuals levels of iron saturation of transferrin are 25% and above; 50% saturation indicates iron overload. Six-minute walk test (6MWT) was reduced in iron-deficient patients compared with iron replete patients, irrespective as to whether they had anemia or not. In a sub-set of patients who received oral iron therapy, only eight out of 18 had ferritin levels restored to normal levels, suggesting impaired oral uptake in these patients (which has subsequently been proposed to be secondary to increased hepcidin levels). These findings were confirmed in a slightly later study where it was also demonstrated that iron deficiency was more prevalent in patients with iPAH compared to patients with chronic thromboembolic PH (CTEPH) (Soon et al., 2011). Interestingly, IL-6 levels were correlated with iron levels in iPAH patients but not in CTEPH patients. As described earlier, IL-6 is known to promote hepcidin production, but in this study there was no correlation of IL-6 with hepcidin in iPAH patients. Data from another United Kingdom group suggested that hepcidin levels may be inappropriately high in a subset of iPAH patients (Rhodes et al., 2011a). Furthermore, iron deficiency, this time defined by increased circulating soluble transferrin receptor levels, was associated with disease severity and poor clinical outcome.

There are also potential implications related to BMPR2 gene mutation or subsequent downstream pathway dysfunction and altered iron handling in PAH, as BMPR2/SMAD pathways, amongst other functions, are also involved in regulation of iron homeostasis, facilitated through the control of hepcidin production (**Figure 2**) (Finberg, 2013). As mentioned earlier, under normal circumstances iron availability directs such homeostatic control. In patients with iPAH this homeostasis is presumably lost, leading to iron deficiency and hepcidin excess (Rhodes et al., 2011b). Inflammation may impact further on dysfunctional BMPR2 signaling and loss of iron homeostasis, as plasma IL-6 levels are raised in patients with PAH (Selimovic et al., 2009); IL-6 is also known to up-regulate hepcidin expression via the JAK-STAT pathway (Soon et al., 2010) (**Figure 2**). Intriguingly, increased autophagy mediated by lysosomal action (where BMPR2 and ferroportin are both degraded) has been implicated in PAH (Long et al., 2013) suggesting a potential link with altered iron handling.

Further evidence for abnormal iron handling was demonstrated by Decker et al. (2011) who showed that zinc protoporphyrin (Zn-pp) levels are high in patients with PAH (mainly iPAH) indicating deficient iron incorporation to form heme suggestive of iron deficiency; levels were closely related to clinical severity (Decker et al., 2011). Zinc competes with iron for binding sites, therefore when iron levels are diminished zinc replaces iron at these sites. PAH patients also had a high red cell distribution width (RDW), again corresponding to markers of clinical severity, such as higher pulmonary arterial pressures and lower 6MWT. Most recently, using proteome analysis in the plasma of patients with PAH, Rhodes et al. (2017) were able to identify a combination of nine circulating proteins associated with a high risk of mortality, two of which, plasminogen and erythropoietin, are associated with abnormal iron metabolism (Rhodes et al., 2017).

### Evidence for Abnormal Iron Handling in Eisenmenger Syndrome

Iron deficiency has been recognized in patients with cyanotic congenital heart disease for much longer than other forms of PAH. This is considered to be a direct response of increased secondary erythrocytosis (due to chronic cyanosis). However, the exact underlying mechanisms remain unclear. An additional factor, historically, was the routine use of venesection, which was associated with increased risk of hemoptysis, anemia and iron deficiency (Daliento et al., 1998). Iron deficiency is associated with adverse outcomes in Eisenmenger patients (Van De Bruaene et al., 2011). Venesection is now not recommended and iron deficiency actively corrected in this population (Dimopoulos et al., 2008).

Although very different diseases, pulmonary vascular remodeling in ES and iPAH is similar; however, dysfunctional BMP signaling linked to the gene defects in BMPR II has not been found in ES (Therrien et al., 2006) (although BMPRII protein levels have been reported to be reduced in this population). As for determinants of disrupted iron homoeostasis in these patients, data are limited but, in a small comparative observational study undertaken by ourselves (published in abstract form), there were significant increases in an array of plasma markers including: soluble transferrin receptor, free heme, iron saturation of transferrin, IL-6 and hepcidin; clearly demonstrating elevated levels of these iron indices in ES cohorts as compared to iPAH patients and healthy controls (Mumby et al., 2016).

### Evidence for Abnormal Iron Handling in Other Forms of PH

#### Group 1: Associated Forms of PAH

With regards to associated forms of PAH in Group 1, only when in association with scleroderma has there been a link with iron deficiency described in the literature. Iron deficiency (as determined by soluble transferrin receptor) was present in 46% of patients with scleroderma-PH compared to 16% of patients with scleroderma but no PH (note that not all of these had PAH). Iron deficiency was associated with lower exercise capacity and worse survival. Hepcidin levels were overall high and related to transferrin receptor status but not to IL-6 levels (Ruiter et al., 2011).

#### Group 3: PH Related to Chronic Lung Disease and/or Hypoxia (Including High Altitude)

As described earlier, iron deficiency causes an exaggerated response, in terms of PASP, to acute hypoxia, compared to iron replete controls. Iron replacement reverses this response (Frise et al., 2016). Apart from periods when humans are exposed to hypoxia, these findings may be relevant to PH that develops in conjunction with respiratory conditions, which are characterized by alveolar hypoxia (Group 3). Indeed, iron deficiency has been shown to associate with echo derived PASP in non-anemic patients with COPD (Plesner et al., 2017). Potential relevant mechanisms are described next.

##### A role for Hif in hypoxia and normoxia

The molecular mechanisms underpinning the response to hypoxia are incompletely understood, although Hif is likely to be key. Mutations which lead to over activation of Hif in normoxia, a condition called Chuvash polycythemia, result in an exaggerated vasoconstrictor response under hypoxic conditions (Smith et al., 2006). Mouse models of the von Hippel-Lindau mutation, that characterizes Chuvash polycythemia, result in PH and fibrosis (Imtiyaz et al., 2010). Moreover, mouse models with heterozygous mutations in Hif components result in some protection from PH under conditions of hypoxia (Yu et al., 1999; Brusselmans et al., 2003). Furthermore, as Hif activity is both regulated by iron and itself regulates aspects of iron homeostatic control, an interactive role for iron and Hif in PH seems plausible. Indeed, for an increasing number of genes involved in iron homeostasis, the presence of IREs and hypoxic responsive elements (HREs) within the promoter region have been described. Expression at the level of mRNA is therefore regulated not just by the IRPs but also by the hypoxia inducible transcription factor component, Hif-2α. Moreover, as mentioned above, Hif-2α activity is controlled via the enzymatic activity of the iron and oxygen sensing prolyl hydroxylases, and depletion of either substrate leads to Hif activation and an array of transcriptional responses. Therefore, iron deficiency, in a similar fashion to hypoxia, can lead to Hif activity; but potentially under normoxic conditions.

##### Hif and regulation of cellular iron content

Importantly, the ferroportin gene contains both IREs and HREs; thus, hypoxia represents a further signal for ferroportin expression via Hif-2α (Simpson and McKie, 2015). As for any role for hypoxia in hepcidin expression/activity there are controversies in the literature with some studies suggesting an involvement for Hif-2α in hepcidin suppression, (Peyssonnaux et al., 2007) whereas more recent literature refutes this suggestion (Volke et al., 2009); however, other soluble factors may be involved (Ravasi et al., 2014). Reactive oxygen species (ROS) may also have a role as the redox sensitive transcription factor, NF-κB, has been shown to regulate hepcidin expression in part in macrophages (Sow et al., 2009). Given the role of hypoxia and ROS production in PAH it is likely that such perturbations will be influential in aspects of disrupted iron homeostasis of relevance to the pulmonary vasculature and will further modulate the hepcidin/ferroportin axis. Another iron homeostatic perturbation that may be of relevance to Hif activity is iron regulatory protein-1 (IRP-1) dysfunction; targeted deletion of IRP-1 in mice resulted in PH and polycythemia, responses, which were more apparent in animals, fed an iron-restricted diet. Hif-2α expression in pulmonary endothelial cells cultured from these animals was elevated in comparison to those from wild type controls (Ghosh et al., 2013); effects on the hepcidin/ferroportin axis were not described in this study. Additionally, iron–sulfur cluster deficiency linked to either hypoxic or genetic alterations in the microRNA-210-ISCU1/2 axis also contribute to PH in murine models, (White et al., 2015) all of which suggests a link to mitochondrial dysfunction, given that iron–sulfur synthesis occurs in this organelle and its function is dramatically affected by ischemia or hypoxia. Finally, there may also be implications in relation to IRP-1 activity which is regulated by iron–sulfur cluster assembly (Kuhn, 2015). The role of Hif, hypoxia and iron as potential drivers of PAH are therefore complex and still emerging.

#### Group 5: Miscellaneous Causes of PH: The Hemolytic Anemias

The last group to mention is the chronic hemolytic anemias, which sit in Group 5 of PH classification. Abnormal iron handling may clearly be a factor in conditions such as Sickle Cell Disease (SCD) and the Thalassemias. Potential factors that may contribute to disease presentation in these circumstances are numerous and include consequences related to the release of free hemoglobin. The potential for a direct proliferative role linked to decompartmentalization of free iron/heme/hemoglobin is also of potential importance and is given consideration below. However, as for mechanisms of disease onset and progression, in addition to the more detailed descriptions presented below, consideration should also be given to high cardiac output-PH in very anemic patients, as well as direct myocardial involvement producing a post-capillary PH picture (Group 2 PH).

##### Hemoglobin and oxidative stress

Hemoglobin, once decompartmentalized, disassociates to the dimeric form, is subject to the release of free heme and is also likely to shed iron, which, in an extracellular setting of complete transferrin iron saturation, will remain free or loosely bound. This, in turn, will be available to catalyze damaging hydroxyl radical formation from hydrogen peroxide and initiate or propagate peroxidation of membrane polyunsaturated fatty acids causing damage that is not necessarily limited to endothelial targets. In addition, both hemoglobin and heme are redox catalysts and so can also contribute to damaging oxidant production and an overall altered redox balance. Thus, there are numerous iron breakdown products released or formed consequent to hemolysis, which may have implications for disease onset and progression.

##### Endogenous protection of a limited resource

Targeted binding by haptoglobin for hemoglobin, hemopexin and or albumin for heme and transferrin or lactoferrin for iron, followed by receptor mediated cellular uptake limits adverse aspects related to the release of these species in the extracellular setting. However, during prolonged or pronounced hemolytic episodes, such binding processes are saturated leading to extracellular accumulation of these products of hemolysis and also increased iron storage within cells and tissues. In this regard, it is now widely recognized that PH is a common co-morbidity for patients suffering from hemoglobinopathies resultant from hemolytic anemias (Souza et al., 2009) and consequently that endogenous protection is compromised in these patient groups.

##### A role for decompartmentalized hemoglobin in PAH

Decompartmentalization of hemoglobin and subsequent binding of NO that affects vascular reactivity and remodeling has been hypothesized as a functional component in PAH associated with hemoglobinopathies such as the Thalassemias and SCD (Mathew et al., 2016). In addition, hemoglobin/heme containing red cell microparticle formation also accompanies hemolysis with known effects on the endothelium and resultant vascular occlusion in SCD (Camus et al., 2015). In epidemiological terms for SCD populations, PAH is many orders of magnitude more common than in the normal population (Buehler et al., 2012) and additionally for SCD patients with PAH, hemolytic anemia is much more severe (Potoka and Gladwin, 2014); all of which strongly suggests active involvement of hemolysis in the development of PAH in SCD. A link which is also apparent for β-thalassemia major where PAH development is thought to be related to both the severity of hemolysis and also the need for repeated transfusions (leading to tissue iron overload); whereas for β-thalassemia intermedia, ongoing hemolysis without transfusion also causes iron loading and is associated with PAH (Fraidenburg and Machado, 2016). In a small study of non-transfusion dependent thalassemia patients, PAH was relatively common (10%) and was associated with previous multiple transfusions, splenectomy and non-transferrin bound iron (a marker of iron overload) (Inthawong et al., 2015). A role in PAH is also apparent for other hemolytic disorders such as hereditary spherocytosis, microangiopathic anemias, and paroxysmal nocturnal hemoglobinuria to name but a few (Machado and Farber, 2013). Importantly, recent studies in patients with iPAH and heritable PAH have demonstrated red cell abnormalities: specifically enhanced levels of zinc-protoporphyrin (Decker et al., 2011); elevated creatine (Fox et al., 2012) and plasma free hemoglobin, which was independently associated with increased risk for admission to hospital (Brittain et al., 2014) all of which are suggestive of iron deficiency and sub-clinical hemolysis. Moreover, decreased levels of haptoglobin are apparent across PAH phenotypes and demonstrates a negative correlation with mean pulmonary artery pressure for patients with connective tissue disease associated PAH (Nakamura et al., 2017). Given the role of haptoglobin as an extracellular hemoglobin binding protein these studies further support a role for hemolysis and decompartmentalized hemoglobin in PAH progression.

## *In Vitro* and *In Vivo* Models of Disrupted Iron Homeostasis

There are a number of recently published studies, which have investigated the relationship between iron availability, or deficiency, and proliferative responses in settings of relevance to PAH. In one study the use of iron chelation via the administration of desferrioxamine to rats was found to inhibit chronic hypoxia induced PH and remodeling suggesting that iron is requisite for vascular proliferation in these circumstances; an assertion further supported by *in vitro* studies by the same authors which showed that an iron chelation strategy also inhibited proliferation of cultured PASMCs (Wong et al., 2012). In another study, use of plumbagin, an iron chelator, (Padhye et al., 2012) was found to limit proliferation in human PASMCs and decrease distal pulmonary artery remodeling in a rat model of PAH (Courboulin et al., 2012). Additionally, iron was found to induce remodeling in cultured rat PAECs (Gorbunov et al., 2012). Together, these studies indicate a role for iron availability in proliferation and remodeling. However, in other recent studies, opposing responses have been reported; for instance, iron deficient (iron restricted diet) monocrotaline treated rats were somewhat protected from pulmonary vascular remodeling and right ventricular failure; however, the low levels of hepcidin shown in these animals compared to controls (iron replete) is at variance with elevated hepcidin as observed in clinical PAH and also suggests that cellular iron retention is less likely in these circumstances (Naito et al., 2013). In a related rat model, iron deficiency alone of 4 weeks duration was shown to contribute to in pulmonary vascular remodeling, raised pulmonary artery pressure and right ventricular hypertrophy. All these changes were reversed on restoration of a dietary iron (Cotroneo et al., 2015) suggesting that iron deficiency contributes to vascular remodeling. Whilst not in absolute agreement, collectively, these studies do indicate that iron availability or otherwise is a key component of vascular remodeling in experimental PAH.

As for any role for iron retention or release at the level of the cell, these studies are fledgling in nature and largely limited to published abstracts. Our own studies in this regard have established that ferroportin is present on both human PAMSCs (Ramakrishnan et al., 2014) and human PAECs (Ramakrishnan et al., 2013) and that hepcidin treatment of human PASMCs causes a proliferative response most likely linked to cellular iron retention. Moreover, treatment with IL-6 also promotes human PASMC proliferation (Ramakrishnan et al., 2015). Interestingly, the hemoglobin-haptoglobin receptor/scavenger molecule, CD163, has been shown to be expressed and regulated in both human PAECs as well as PASMCs (Ramakrishnan et al., 2015, 2016) suggesting a means for cellular uptake of iron by these cells. In related studies, an *in vivo* model of vascular remodeling linked to cellular iron accumulation was reversed with haptoglobin therapy (Irwin et al., 2015) indicating a potential role for free hemoglobin in these processes. Thus there may be a generalized functional impact related to decompartmentalization of hemoglobin of relevance to PAH which given our observational data (Mumby et al., 2016) may be of greater relevance in condition such as Eisenmenger syndrome.

## Treatments: Summary of Clinical Evidence With Iron Replacement in Pah

There are very few clinical trials investigating the effect of iron replacement in patients with PAH. Viethen et al. (2014) presented the results of 20 iron deficient patients with mixed PAH etiology, given IV ferric carboxymaltose (1,000 mg) in an open label fashion. Compared to a non-treated group, there was an improvement in iron status, significant increase in 6-min walk test and quality of life score (Viethen et al., 2014). There have been two trials in specific PAH sub-types, described below.

### Iron Therapy in Cyanotic Congenital Heart Disease

There are no randomized, placebo controlled studies investigating the effect of iron replacement in the cyanotic congenital heart disease population. However, Tay et al. (2011) studied 25 iron-deficient cyanotic CHD patients, 14 of which had Eisenmenger Syndrome, who received oral iron replacement in a prospective open label manner (Tay et al., 2011). Oral ferrous fumarate was titrated to a maximum of 200 mg tds. After 3 months of treatment, hemoglobin, ferritin and transferrin saturation had all significantly increased. Significant improvements were also seen in quality of life and 6MWT, although peak VO_2_ was unchanged. Oral treatment was well tolerated with no complications. In clinical practice in the cyanotic CHD population treatment of iron deficiency (as a complication of secondary erythrocytosis) is managed quite aggressively, usually with intravenous iron replacement. In 142 consecutive cyanotic CHD patients of which 49% were ES, treatment with IV ferrous carboxymaltose (500 mg; Ferrinject^TM^) resulted in improvement in hemoglobin, hematocrit, ferritin, and transferrin saturation. There were no cases of excessive erythrocytosis with very few complications (personal communication).

### Iron Therapy and Idiopathic Pulmonary Arterial Hypertension

There is only one published study on the use of iron replacement in patients with iPAH (Gerrina et al., 2015). Fifteen patients with iPAH and iron deficiency received a single, high dose of IV iron in an open label fashion. After 12 weeks, the primary endpoint, 6MWT, was not changed. However, exercise endurance time and aerobic capacity increased significantly without change in cardiac function. There was improved quadriceps muscle oxygen handling. IV iron was well tolerated and there was an improvement in quality of life. A randomized, placebo controlled trial is currently active and the results are eagerly awaited (Howard et al., 2013).

### Iron Therapy in Other Forms of PH

There are no other studies investigating the use of iron replacement in other forms of PH apart from experimental studies in acute hypoxia driven PH. Bart et al. (2016) investigated the effect of 1 g IV iron (or saline) on increases in PASP associated with 6 h of hypoxia in 22 subjects. Patients receiving iron before hypoxia had a 50% reduction in PASP rise which was sustained up to 43 days after iron therapy (Bart et al., 2016).

### Future Treatments

Clearly from the literature presented above, it is apparent that protocols are under development and in use involving iron supplementation therapies in order to correct perceived iron patient deficits. Whilst this ultimately may help restore iron homeostasis there is nevertheless the potential to for cellular iron-loading to occur particularly in a setting of hepcidin excess as is seen in PAH; in which case shorter term benefits may be outweighed by longer term effects on pulmonary vascular remodeling. Other potential solutions that would aim to restore iron homeostatic control could involve targeted inhibition (modulation) of the hepcidin/ferroportin axis. In fact, there are several treatment options under development/developed which either seek to stabilize ferroportin expression and activity in spite of hepcidin excess or that target hepcidin directly (Sebastiani et al., 2016). Such approaches will thereby allow for iron uptake from the gut and also cellular iron excretion, which may be of relevance for the pulmonary vasculature and any links with iron retention and remodeling in PAH. Finally, given the relationship between IL-6 and hepcidin expression and release, therapies that selectively target this cytokine would also be expected to influence iron homeostatic control. Indeed such approaches are already underway in PAH groups (TRANSFORM UK study) albeit not specifically to correct iron imbalance.

## Summary

Presently the field of disrupted iron homeostasis in PAH is poorly understood, not least because most studies of iron regulatory control have centered on global mechanisms and cells principally involved in recycling body iron resources.

However, emerging literature does suggest similar regulatory controls are also operational across other cell types including, potentially, in the vasculature. Links between disrupted iron homeostatic control and sub-types of PH, especially PAH, PH associated to hemolytic anemias and hypoxia may therefore well have implications for iron turnover and control in pulmonary artery vascular cells. Debate centered on the role of iron deficits or overload in PAH and models thereof may merely serve to strengthen the supposition that loss of iron homeostatic control is an important contributory factor to disease onset and progression. Additional studies may well confirm these suggestions and ultimately offer much needed alternative therapeutic options.

## Author Contributions

SW and GQ were responsible for the conception of idea and design and wrote most of the manuscript. LR was responsible for the contribution of ideas, design, figures, and copy-editing. SP was responsible for the contribution of ideas, figures, and contents research. QT contributed ideas, copy-editing, and contents research. All authors contributed to the manuscript revision, read and approved the final version.

## Conflict of Interest Statement

The authors declare that the research was conducted in the absence of any commercial or financial relationships that could be construed as a potential conflict of interest.

## References

[B1] Barst R. J. Rubin L. J. Long W. A. Mcgoon M. D. Rich S. Badesch D. B. (1996). A comparison of continuous intravenous Epoprostenol (Prostacyclin) with conventional therapy for primary pulmonary hypertension. *N. Engl. J. Med.* 334 296–301. 10.1056/NEJM199602013340504 8532025

[B2] Bart N. K. Curtis M. K. Cheng H.-Y. Hungerford S. L. Mclaren R. Petousi N. (2016). Elevation of iron storage in humans attenuates the pulmonary vascular response to hypoxia. *J. Appl. Physiol.* 121 537–544. 10.1152/japplphysiol.00032.2016 27418684 PMC5007321

[B3] Brittain E. L. Janz D. R. Austin E. D. Bastarache J. A. Wheeler L. A. Ware L. B. (2014). Elevation of plasma cell-free hemoglobin in pulmonary arterial hypertension. *Chest* 146 1478–1485. 10.1378/chest.14-0809 24945582 PMC4251612

[B4] Brusselmans K. Compernolle V. Tjwa M. Wiesener M. S. Maxwell P. H. Collen D. (2003). Heterozygous deficiency of hypoxia-inducible factor–2α protects mice against pulmonary hypertension and right ventricular dysfunction during prolonged hypoxia. *J. Clin. Invest.* 111 1519–1527. 10.1172/JCI15496 12750401 PMC155039

[B5] Buehler P. W. Baek J. H. Lisk C. Connor I. Sullivan T. Kominsky D. (2012). Free hemoglobin induction of pulmonary vascular disease: evidence for an inflammatory mechanism. *Am. J. Physiol. Lung Cell. Mol. Physiol.* 303 L312–L326. 10.1152/ajplung.00074.2012 22728465 PMC3423829

[B6] Camus S. M. De Moraes J. A. Bonnin P. Abbyad P. Le Jeune S. Lionnet F. (2015). Circulating cell membrane microparticles transfer heme to endothelial cells and trigger vasoocclusions in sickle cell disease. *Blood* 125 3805–3814. 10.1182/blood-2014-07-589283 25827830 PMC4490297

[B7] Cotroneo E. Ashek A. Wang L. Wharton J. Dubois O. Bozorgi S. (2015). Iron homeostasis and pulmonary hypertension: iron deficiency leads to pulmonary vascular remodeling in the rat. *Circ. Res.* 116 1680–1690. 10.1161/CIRCRESAHA.116.305265 25767292

[B8] Courboulin A. Barrier M. Perreault T. Bonnet P. Tremblay V. L. Paulin R. (2012). Plumbagin reverses proliferation and resistance to apoptosis in experimental Pah. *Eur. Respir. J.* 40 618–629. 10.1183/09031936.00084211 22496325

[B9] Daliento L. Somerville J. Presbitero P. Menti L. Brach-Prever S. Rizzoli G. (1998). Eisenmenger syndrome. Factors relating to deterioration and death. *Eur. Heart J.* 19 1845–1855. 10.1053/euhj.1998.1046 9886728

[B10] D’Alto M. Mahadevan V. S. (2012). Pulmonary arterial hypertension associated with congenital heart disease. *Eur. Respir. Rev.* 21 328–337. 10.1183/09059180.00004712 23204121 PMC9487226

[B11] Decker I. Ghosh S. Comhair S. A. Farha S. Tang W. H. Park M. (2011). High levels of zinc-protoporphyrin identify iron metabolic abnormalities in pulmonary arterial hypertension. *Clin. Transl. Sci.* 4 253–258. 10.1111/j.1752-8062.2011.00301.x 21884511 PMC3575639

[B12] Dimopoulos K. Giannakoulas G. Wort S. J. Gatzoulis M. A. (2008). Pulmonary arterial hypertension in adults with congenital heart disease: distinct differences from other causes of pulmonary arterial hypertension and management implications. *Curr. Opin. Cardiol.* 23 545–554. 10.1097/HCO.0b013e3283126954 18830068

[B13] Drakesmith H. Nemeth E. Ganz T. (2015). Ironing out Ferroportin. *Cell Metab.* 22 777–787. 10.1016/j.cmet.2015.09.006 26437604 PMC4635047

[B14] Finberg K. E. (2013). Regulation of systemic iron homeostasis. *Curr. Opin. Hematol.* 20 208–214. 10.1097/MOH.0b013e32835f5a47 23426198

[B15] Fox B. D. Okumiya T. Attas-Fox L. Kassirer M. Raviv Y. Kramer M. R. (2012). Raised erythrocyte creatine in patients with pulmonary arterial hypertension–evidence for subclinical hemolysis. *Respir. Med.* 106 594–598. 10.1016/j.rmed.2011.12.005 22244549

[B16] Fraidenburg D. R. Machado R. F. (2016). Pulmonary hypertension associated with thalassemia syndromes. *Ann. N. Y. Acad. Sci.* 1368 127–139. 10.1111/nyas.13037 27008311 PMC4870173

[B17] Frise M. C. Cheng H.-Y. Nickol A. H. Curtis M. K. Pollard K. A. Roberts D. J. (2016). Clinical iron deficiency disturbs normal human responses to hypoxia. *J. Clin. Invest.* 126 2139–2150. 10.1172/JCI85715 27140401 PMC4887172

[B18] Galie N. Humbert M. Vachiery J. L. Gibbs S. Lang I. Torbicki A. (2016). 2015 ESC/ERS guidelines for the diagnosis and treatment of pulmonary hypertension: the joint task force for the diagnosis and treatment of pulmonary hypertension of the European society of cardiology (ESC) and the European respiratory society (ERS): endorsed by: association for European paediatric and congenital cardiology (AEPC), international society for heart and lung transplantation (ISHLT). *Eur. Heart J.* 37 67–119. 10.1093/eurheartj/ehv317 26320113

[B19] Gerrina R. Emmy M. Chris M. H. Ingrid S. Herman G. Luke S. H. (2015). Intravenous iron therapy in patients with idiopathic pulmonary arterial hypertension and iron deficiency. *Pulm. Circ.* 5 466–472. 10.1086/682217 26401247 PMC4556497

[B20] Ghosh M. C. Zhang D. L. Jeong S. Y. Kovtunovych G. Ollivierre-Wilson H. Noguchi A. (2013). Deletion of iron regulatory protein 1 causes polycythemia and pulmonary hypertension in mice through translational derepression of HIF2ALPHA. *Cell Metab.* 17 271–281. 10.1016/j.cmet.2012.12.016 23395173 PMC3569856

[B21] Gorbunov N. V. Atkins J. L. Gurusamy N. Pitt B. R. (2012). Iron-induced remodeling in cultured rat pulmonary artery endothelial cells. *Biometals* 25 203–217. 10.1007/s10534-011-9498-2 22089858

[B22] Howard L. S. Watson G. M. Wharton J. Rhodes C. J. Chan K. Khengar R. (2013). Supplementation of iron in pulmonary hypertension: Rationale and design of a phase II clinical trial in idiopathic pulmonary arterial hypertension. *Pulm. Circ.* 3 100–107. 10.4103/2045-8932.109923 23662181 PMC3641712

[B23] Humbert M. Sitbon O. Chaouat A. Bertocchi M. Habib G. Gressin V. (2006). Pulmonary arterial hypertension in France. *Am. J. Respir. Crit. Care Med.* 173 1023–1030. 10.1164/rccm.200510-1668OC 16456139

[B24] Humbert M. Sitbon O. Simonneau G. (2004). Treatment of pulmonary arterial hypertension. *N. Engl. J. Med.* 351 1425–1436. 10.1056/NEJMra040291 15459304

[B25] Imtiyaz H. Z. Williams E. P. Hickey M. M. Patel S. A. Durham A. C. Yuan L.-J. (2010). Hypoxia-inducible factor 2α regulates macrophage function in mouse models of acute and tumor inflammation. *J. Clin. Invest.* 120 2699–2714. 10.1172/JCI39506 20644254 PMC2912179

[B26] Inthawong K. Charoenkwan P. Silvilairat S. Tantiworawit A. Phrommintikul A. Choeyprasert W. (2015). Pulmonary hypertension in non-transfusion-dependent thalassemia: correlation with clinical parameters, liver iron concentration, and non-transferrin-bound iron. *Hematology* 20 610–617. 10.1179/1607845415Y.0000000014 25964094

[B27] Irwin D. C. Baek J. H. Hassell K. Nuss R. Eigenberger P. Lisk C. (2015). Hemoglobin induced lung vascular oxidation, inflammation, and remodeling contributes to the progression of hypoxic pulmonary hypertension and is attenuated in rats with repeat dose haptoglobin administration. *Free Radic. Biol. Med.* 82 50–62. 10.1016/j.freeradbiomed.2015.01.012 25656991 PMC4387123

[B28] Kuhn L. C. (2015). Iron regulatory proteins and their role in controlling iron metabolism. *Metallomics* 7 232–243. 10.1039/C4MT00164H 25306858

[B29] Long L. Yang X. Southwood M. Lu J. Marciniak S. J. Dunmore B. J. (2013). Chloroquine prevents progression of experimental pulmonary hypertension via inhibition of autophagy and lysosomal bone morphogenetic protein type II receptor degradation. *Circ. Res.* 112 1159–1170. 10.1161/CIRCRESAHA.111.300483 23446737

[B30] Machado R. F. Farber H. W. (2013). Pulmonary hypertension associated with chronic hemolytic anemia and other blood disorders. *Clin. Chest Med.* 34 739–752. 10.1016/j.ccm.2013.08.006 24267302 PMC3916937

[B31] Mathew R. Huang J. Wu J. M. Fallon J. T. Gewitz M. H. (2016). Hematological disorders and pulmonary hypertension. *World J. Cardiol.* 8 703–718. 10.4330/wjc.v8.i12.703 28070238 PMC5183970

[B32] Mumby S. Aleksander Kempny L. R. Quinlan G. Wort J. (2016). Omalizumab, airway obstruction and remodeling. *Eur. Respir. J.* 48:PA4901 10.1183/13993003.congress-2015.PA4901

[B33] Naito Y. Hosokawa M. Hao H. Sawada H. Hirotani S. Iwasaku T. (2013). Impact of dietary iron restriction on the development of monocrotaline-induced pulmonary vascular remodeling and right ventricular failure in rats. *Biochem. Biophys. Res. Commun.* 436 145–151. 10.1016/j.bbrc.2013.05.059 23707944

[B34] Nakamura H. Kato M. Nakaya T. Kono M. Tanimura S. Sato T. (2017). Decreased haptoglobin levels inversely correlated with pulmonary artery pressure in patients with pulmonary arterial hypertension: a cross-sectional study. *Medicine* 96:e8349. 10.1097/MD.0000000000008349 29069014 PMC5671847

[B35] Padhye S. Dandawate P. Yusufi M. Ahmad A. Sarkar F. H. (2012). Perspectives on medicinal properties of plumbagin and its analogs. *M*ed. Res. Rev.32 1131–1158. 10.1002/med.20235 23059762

[B36] Peacock A. J. Murphy N. F. Mcmurray J. J. V. Caballero L. Stewart S. (2007). An epidemiological study of pulmonary arterial hypertension. *Eur. Respir. J.* 30 104–109. 10.1183/09031936.00092306 17360728

[B37] Peyssonnaux C. Zinkernagel A. S. Schuepbach R. A. Rankin E. Vaulont S. Haase V. H. (2007). Regulation of iron homeostasis by the hypoxia-inducible transcription factors (HIFS). *J. Clin. Invest.* 117 1926–1932. 10.1172/JCI31370 17557118 PMC1884690

[B38] Plesner L. L. Schoos M. M. Dalsgaard M. Goetze J. P. Kjøller E. Vestbo J. (2017). Iron deficiency in Copd associates with increased pulmonary artery pressure estimated by echocardiography. *Heart Lung Circ.* 26 101–104. 10.1016/j.hlc.2016.04.020 27372430

[B39] Potoka K. P. Gladwin M. T. (2014). Vasculopathy and pulmonary hypertension in sickle cell disease. *Am. J. Physiol. Lung Cell. Mol. Physiol.* 308 L314–L324. 10.1152/ajplung.00252.2014 25398989 PMC4329471

[B40] Pullamsetti S. S. Perros F. Chelladurai P. Yuan J. Stenmark K. (2016). Transcription factors, transcriptional coregulators, and epigenetic modulation in the control of pulmonary vascular cell phenotype: therapeutic implications for pulmonary hypertension (2015 Grover Conference series). *Pulm. Circ.* 6 448–464. 10.1086/688908 28090287 PMC5210074

[B41] Ramakrishnan L. Anwar A. Wort J. S. Quinlan G. J. (2016). Haemoglobin mediated proliferation and Il-6 release in human pulmonary artery endothelial cells: a role for Cd163 and implications for pulmonary vascular remodelling. *Thorax* 71 A220–A220. 10.1136/thoraxjnl-2016-209333.387

[B42] Ramakrishnan L. Mumby S. Meng C. Wort S. J. Quinlan G. (2013). IL6 mediated proliferative responses in human pulmonary vascular cells (PVCs) are differentially modulated by iron/heme/hemoglobin. *Eur. Respir. J.* 42:P5152.

[B43] Ramakrishnan L. Mumby S. Wort J. S. Quinlan G. (2014). Ferroportin is expressed in human pulmonary artery smooth muscle cells: implications for pulmonary arterial hypertension. *Thorax* 69 A21–A21. 10.1136/thoraxjnl-2014-206260.42

[B44] Ramakrishnan L. Mumby S. Wort J. S. Quinlan G. J. (2015). Cd163 is expressed and modulated in human pulmonary artery smooth muscle cells: Implications for pulmonary artery hypertension. *Eur. Respir. J.* 46:PA4901 10.1183/13993003.congress-2015.PA4901

[B45] Ravasi G. Pelucchi S. Greni F. Mariani R. Giuliano A. Parati G. (2014). Circulating factors are involved in hypoxia-induced hepcidin suppression. *Blood Cells Mol. Dis.* 53 204–210. 10.1016/j.bcmd.2014.06.006 25065484

[B46] Rhodes C. J. Howard L. S. Busbridge M. Ashby D. Kondili E. Gibbs J. S. R. (2011a). Iron deficiency and raised hepcidin in idiopathic pulmonary arterial hypertension: clinical prevalence, outcomes, and mechanistic insights. *J. Am. Coll. Cardiol.* 58 300–309. 10.1016/j.jacc.2011.02.057 21737024

[B47] Rhodes C. J. Wharton J. Howard L. Gibbs J. S. Vonk-Noordegraaf A. Wilkins M. R. (2011b). Iron deficiency in pulmonary arterial hypertension: a potential therapeutic target. *Eur. Respir. J.* 38 1453–1460. 10.1183/09031936.00037711 21478213

[B48] Rhodes C. J. Wharton J. Ghataorhe P. Watson G. Girerd B. Howard L. S. (2017). Plasma proteome analysis in patients with pulmonary arterial hypertension: an observational cohort study. *Lancet Respir. Med.* 5 717–726. 10.1016/S2213-2600(17)30161-3 28624389 PMC5573768

[B49] Rudarakanchana N. Flanagan J. A. Chen H. Upton P. D. Machado R. Patel D. (2002). Functional analysis of bone morphogenetic protein type II receptor mutations underlying primary pulmonary hypertension. *Hum. Mol. Genet.* 11 1517–1525. 10.1093/hmg/11.13.1517 12045205

[B50] Ruiter G. Lankhorst S. Boonstra A. Postmus P. E. Zweegman S. Westerhof N. (2011). Iron deficiency is common in idiopathic pulmonary arterial hypertension. *Eur. Respir. J.* 37 1386–1391. 10.1183/09031936.00100510 20884742

[B51] Sebastiani G. Wilkinson N. Pantopoulos K. (2016). Pharmacological Targeting of the Hepcidin/Ferroportin Axis. *Front. Pharmacol.* 7:160. 10.3389/fphar.2016.00160 27445804 PMC4914558

[B52] Selimovic N. Bergh C. H. Andersson B. Sakiniene E. Carlsten H. Rundqvist B. (2009). Growth factors and interleukin-6 across the lung circulation in pulmonary hypertension. *Eur. Respir. J.* 34 662–668. 10.1183/09031936.001 19324949

[B53] Simpson R. J. McKie A. T. (2015). Iron and oxygen sensing: a tale of 2 interacting elements? *Metallomics* 7 223–231. 10.1039/C4MT00225C 25385426

[B54] Smith T. G. Balanos G. M. Croft Q. P. P. Talbot N. P. Dorrington K. L. Ratcliffe P. J. (2008). The increase in pulmonary arterial pressure caused by hypoxia depends on iron status. *J. Physiol.* 586 5999–6005. 10.1113/jphysiol.2008.160960 18955380 PMC2655431

[B55] Smith T. G. Brooks J. T. Balanos G. M. Lappin T. R. Layton D. M. Leedham D. L. (2006). Mutation of von Hippel–Lindau tumour suppressor and human cardiopulmonary physiology. *PLoS Med.* 3:e290. 10.1371/journal.pmed.0030290 16768548 PMC1479389

[B56] Smith T. G. Talbot N. P. Privat C. (2009). Effects of iron supplementation and depletion on hypoxic pulmonary hypertension: Two randomized controlled trials. *JAMA* 302 1444–1450. 10.1001/jama.2009.1404 19809026

[B57] Soon E. Holmes A. M. Treacy C. M. Doughty N. J. Southgate L. Machado R. D. (2010). Elevated levels of inflammatory cytokines predict survival in idiopathic and familial pulmonary arterial hypertension. *Circulation* 122 920–927. 10.1161/CIRCULATIONAHA.109.933762 20713898

[B58] Soon E. Treacy C. M. Toshner M. R. Mackenzie-Ross R. Manglam V. Busbridge M. (2011). Unexplained iron deficiency in idiopathic and heritable pulmonary arterial hypertension. *Thorax* 66 326–332. 10.1136/thx.2010.147272 21297151

[B59] Soubrier F. Chung W. K. Machado R. Grünig E. Aldred M. Geraci M. (2013). Genetics and Genomics of Pulmonary Arterial Hypertension. *J. Am. Coll. Cardiol.* 62 D13–D21. 10.1016/j.jacc.2013.10.035 24355637

[B60] Souza R. Fernandes C. J. Jardim C. V. (2009). Other causes of PAH (schistosomiasis, porto-pulmonary hypertension and hemolysis-associated pulmonary hypertension). *Semin. Respir. Crit. Care Med.* 30 448–457. 10.1055/s-0029-1233314 19634084

[B61] Sow F. B. Alvarez G. R. Gross R. P. Satoskar A. R. Schlesinger L. S. Zwilling B. S. (2009). Role of STAT1, NF-kappaB, and C/EBPbeta in the macrophage transcriptional regulation of hepcidin by mycobacterial infection and Ifn-gamma. *J. Leukoc. Biol.* 86 1247–1258. 10.1189/jlb.1208719 19652026

[B62] Tay E. L. Peset A. Papaphylactou M. Inuzuka R. Alonso-Gonzalez R. Giannakoulas G. (2011). Replacement therapy for iron deficiency improves exercise capacity and quality of life in patients with cyanotic congenital heart disease and/or the Eisenmenger syndrome. *Int. J. Cardiol.* 151 307–312. 10.1016/j.ijcard.2010.05.066 20580108

[B63] Therrien J. Rambihar S. Newman B. Siminovitch K. Langleben D. Webb G. (2006). Eisenmenger syndrome and atrial septal defect: nature or nurture? *Can. J. Cardiol.* 22 1133–1136. 10.1016/S0828-282X(06)70950-3 17102831 PMC2569052

[B64] Tuder R. M. Archer S. L. Dorfmüller P. Erzurum S. C. Guignabert C. Michelakis E. (2013). Relevant issues in the pathology and pathobiology of pulmonary hypertension. *J. Am. Coll. Cardiol.* 62 D4–D12. 10.1016/j.jacc.2013.10.025 24355640 PMC3970402

[B65] Van De Bruaene A. Delcroix M. Pasquet A. De Backer J. De Pauw M. Naeije R. (2011). Iron deficiency is associated with adverse outcome in Eisenmenger patients. *Eur. Heart J.* 32 2790–2799. 10.1093/eurheartj/ehr130 21606083

[B66] Viethen T. Gerhardt F. Dumitrescu D. Knoop-Busch S. Ten Freyhaus H. Rudolph T. K. (2014). Ferric carboxymaltose improves exercise capacity and quality of life in patients with pulmonary arterial hypertension and iron deficiency: a pilot study. *Int. J. Cardiol.* 175 233–239. 10.1016/j.ijcard.2014.04.233 24880481

[B67] Volke M. Gale D. P. Maegdefrau U. Schley G. Klanke B. Bosserhoff A. K. (2009). Evidence for a lack of a direct transcriptional suppression of the iron regulatory peptide hepcidin by hypoxia-inducible factors. *PLoS One* 4:e7875. 10.1371/journal.pone.0007875 19924283 PMC2773926

[B68] White K. Lu Y. Annis S. Hale A. E. Chau B. N. Dahlman J. E. (2015). Genetic and hypoxic alterations of the microrna-210-Iscu1/2 axis promote iron–sulfur deficiency and pulmonary hypertension. *EMBO Mol. Med.* 7 695–713. 10.15252/emmm.201404511 25825391 PMC4459813

[B69] Wilkins M. R. Ghofrani H. A. Weissmann N. Aldashev A. Zhao L. (2015). Pathophysiology and treatment of high-altitude pulmonary vascular disease. *Circulation* 131 582–590. 10.1161/CIRCULATIONAHA.114.006977 25666980

[B70] Wong C. M. Preston I. R. Hill N. S. Suzuki Y. J. (2012). Iron chelation inhibits the development of pulmonary vascular remodeling. *Free Radic. Biol. Med.* 53 1738–1747. 10.1016/j.freeradbiomed.2012.08.576 22974762 PMC3472156

[B71] Yang X. Long L. Reynolds P. N. Morrell N. W. (2011). Expression of mutant BMPR-II in pulmonary endothelial cells promotes apoptosis and a release of factors that stimulate proliferation of pulmonary arterial smooth muscle cells. *Pulm. Circ.* 1 103–110. 10.4103/2045-8932.78100 22034596 PMC3198633

[B72] Yu A. Y. Shimoda L. A. Iyer N. V. Huso D. L. Sun X. Mcwilliams R. (1999). Impaired physiological responses to chronic hypoxia in mice partially deficient for hypoxia-inducible factor 1α. *J. Clin. Invest.* 103 691–696. 10.1172/JCI5912 10074486 PMC408131

[B73] Yu Y. Kovacevic Z. Richardson D. R. (2007). Tuning cell cycle regulation with an iron key. *Cell Cycle* 6 1982–1994. 10.4161/cc.6.16.4603 17721086

